# CHADS_2_, CHA_2_DS_2_ASc, and New ABCD Scores Predict the Risk of Peripheral Arterial Disease in Patients with Sleep Apnea

**DOI:** 10.3390/jcm8020188

**Published:** 2019-02-05

**Authors:** Kuan-Li Wu, Chia-Yu Kuo, Yu-Chen Tsai, Jen-Yu Hung, Chau-Chyun Sheu, Chih-Jen Yang, Chung-Yao Hsu, Meng-Ni Wu, Ming-Ju Tsai

**Affiliations:** 1 Division of Pulmonary and Critical Care Medicine, Department of Internal Medicine, Kaohsiung Medical University Hospital, Kaohsiung Medical University, Kaohsiung 807, Taiwan; 980448KMUH@gmail.com (K.-L.W.); goba2356@gmail.com (C.-Y.K.); 1010362KMUH@gmail.com (Y.-C.T.); jenyuhung@gmail.com (J.-Y.H.); sheucc@gmail.com (C.-C.S.); chjeya@kmu.edu.tw (C.-J.Y.); 2 Department of Internal Medicine, Kaohsiung Municipal Ta-Tung Hospital, Kaohsiung Medical University, Kaohsiung 807, Taiwan; 3 Sleep Disorders Center, Kaohsiung Medical University Hospital, Kaohsiung Medical University, Kaohsiung 807, Taiwan; cyhsu61@gmail.com (C.-Y.H.); berkeley114@yahoo.com.tw (M.-N.W.); 4 Graduate Institute of Medicine, College of Medicine, Kaohsiung Medical University, Kaohsiung 807, Taiwan; 5 School of Medicine, College of Medicine, Kaohsiung Medical University, Kaohsiung 807, Taiwan; 6 Department of Respiratory Care, College of Medicine, Kaohsiung Medical University, Kaohsiung 807, Taiwan; 7 Graduate Institute of Clinical Medicine, College of Medicine, Kaohsiung Medical University, Kaohsiung 807, Taiwan; 8 Department of Neurology, Kaohsiung Medical University Hospital, Kaohsiung Medical University, Kaohsiung 807, Taiwan

**Keywords:** CHADS_2_ score, CHA_2_DS_2_-VASc score, sleep apnea, peripheral artery disease, sleep disordered breathing

## Abstract

The association between sleep apnea (SA) and peripheral artery disease (PAD) remains debatable, and there is no clinical tool to predict incident PAD in SA patients. The CHADS_2_ score has been found useful in predicting PAD risk. This study was designed to investigate the association between these diseases and the usefulness of CHADS_2_ and CHA_2_DS_2_ASc scores in predicting subsequent PAD in SA patients. From a population-based database of one-million representative subjects, adult patients with SA diagnosis were enrolled as the suspected SA group, and those having SA diagnosis after polysomnography were further extracted as the probable SA group. Twenty sex- and age-matched control subjects were randomly selected for each SA patients. The occurrence of PAD after SA was taken as the primary endpoint. Totally, 10,702 and 4242 patients were enrolled in the suspected and probable SA groups, respectively. The cumulative incidence of PAD was similar between SA patients and the corresponding control groups. Multivariable Cox regression analyses showed that SA was not an independent risk factor for subsequent PAD. Sensitivity analyses using propensity score-matched cohorts showed consistent results. Furthermore, in stratifying the SA patients by CHADS_2_, CHA_2_DS_2_ASc, or a newly-proposed ABCD (composed of Age, high Blood pressure, Cerebral vascular disease, and Diabetes mellitus) score, patients with higher scores predicted higher risks of subsequent PAD, while the ABCD score appeared to be the most robust. Aggressive risk modification is suggested to reduce the subsequent PAD risk in SA patients with a higher CHADS_2_, CHA_2_DS_2_ASc, or ABCD score.

## 1. Introduction

Sleep apnea (SA) is a type of sleep disorder that is characterized by repetitive cessations of respiration during sleep. Polysomnography (PSG) is usually required to comprehensively confirm the diagnosis. Over 90% patients of SA exhibit the obstructive form, namely obstructive sleep apnea (OSA), which is characterized by reduced inspiratory airflow due to upper airway obstruction [1]. The estimated prevalence of OSA is 15% in men and 5% in women between the ages of 30–70 years [2]. SA has multi-dimensional effects on neuropsychiatric, metabolic, and cardiovascular systems. Among these, the impact on cardiovascular diseases, such as hypertension, coronary artery disease, stroke, heart failure, and arrhythmias, has drawn much attention and it is studied broadly [3].

Peripheral artery disease (PAD) is currently the preferred term to describe partial or total occlusion of peripheral arteries secondary to atherosclerosis. Its prevalence increases by age and it is estimated to be around 10% in adults older than 55 years [4]. PAD comprises a wide range of clinical presentations from asymptomatic lesions to critical limb ischemia needing percutaneous or surgical revascularization. PAD shares similar risk factors with other common cardiovascular or cerebrovascular diseases, including cigarette smoking, diabetes mellitus, hypertension, dyslipidemia, chronic kidney disease (CKD), obesity, and so on. In a recent meta-analysis, increased risk of PAD was observed in patients with mild-to-moderate CKD [5]. Patients with PAD also have increased morbidity and mortality of incident coronary and cerebrovascular diseases [6].

Similar to other cardiovascular diseases, the association between SA and PAD has been discussed in the literature. A German study showed higher prevalence of PAD in OSA patients [7]. Chen et al. showed SA as a risk factor for PAD, but the effect became insignificant after adjusting for comorbidities [8]. Furthermore, there is no useful model in predicting the subsequent risk of PAD in SA patients currently. This may hamper and delay appropriate management, leading to worse clinical outcomes.

The CHADS_2_ score has been developed to be a simple and reliable clinical scoring system for assessing the risk of future stroke in patients with atrial fibrillation [9]. Previous studies further showed its usefulness in predicting newly-onset PAD events in patients without atrial fibrillation [10,11]. As SA has already been associated with cardiovascular diseases, whether these findings are applicable to SA patients remains uncertain.

In this nationwide population-based study using Taiwan National Health Insurance Research Database (NHIRD), we investigated the association between PAD and SA, as well as the usefulness of the CHADS_2_ and CHA_2_DS_2_ASc score for the risk of subsequent PAD in SA patients.

## 2. Materials and Methods

### 2.1. Data Sources

The Taiwan National Health Insurance (NHI) has covered ambulatory care, inpatient care, and prescription drugs since March, 1995 and has covered more than 96% of the whole population of 23 million in 2000 [12,13,14]. An NHI research database (NHIRD), containing all medical reimbursement claims, was managed and released by the National Health Research Institutes in Taiwan for research. This study used the Longitudinal Health Insurance Database 2005 (LHID2005), a cohort of one-million randomly sampled subjects in the NHI system in 2005, which included the reimbursement information until the end of 2013. The identification of patients, doctors, and hospitals was encrypted for confidentiality. The Institutional Review Board in Kaohsiung Medical University Hospital approved the study (KMUH-IRB-EXEMPT-20130034).

### 2.2. Study Population

**Figure 1:**
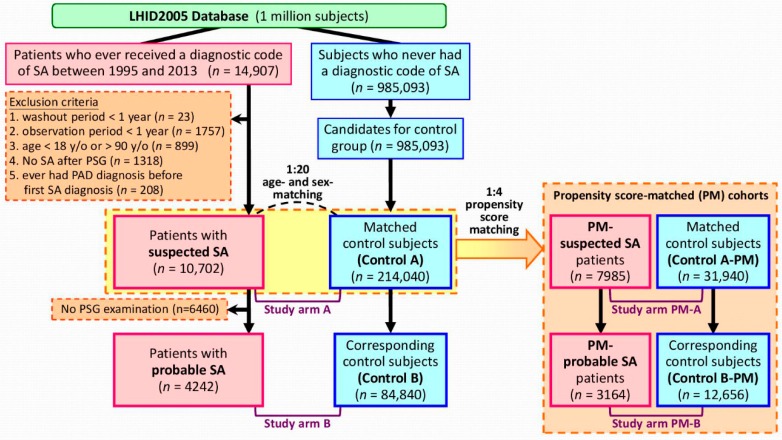
Algorithm for identifying the study population. Abbreviations: SA = sleep apnea; PAD = peripheral arterial disease.

The method for enrolling study subjects is shown in Figure 1. Patients with SA diagnosis between March 1, 1995 and December 31, 2013 were identified by using the International Classification of Diseases, Ninth Revision, Clinical Modification (ICD-9-CM) codes of 780.51, 780.53, and 780.57, as validated in previous studies [13,15,16,17,18,19]. The dates of their first SA diagnosis were defined as their index dates. In order to increase the likelihood of including only newly diagnosed SA cases and ensuring enough observation times, patients with washout periods (from NHI enrollment to the index date) of less than a year or follow-up periods less than a year were excluded, as were patients younger than 18 years or older than 90 years on the index date. Patients who had ever had a PSG examination but had no SA diagnosis after the exam were excluded, as were patients having PAD before the index date. Finally, we defined the remaining patients as the “suspected SA” cohort, because the diagnosis might be made only based on the clinical diagnosis. Among these patients, those having SA diagnosis after PSG examination were extracted as the “probable SA” cohort, because the diagnosis might be confirmed by PSG examination.

For each SA patient, twenty age- and sex-matched control subjects were randomly selected. The same index date as the corresponding SA patient was given to each control subject. During the matching processes, the same exclusion criteria for the SA patients were also applied while selecting the control subjects to ensure enough washout periods and follow-up periods, and the absence of PAD diagnosis before the index date.

### 2.3. Study Outcome

The endpoint of this study was the development of PAD, as defined by the first appearance of PAD diagnosis. The ICD-9-CM codes of 440.2, 440.3, 443.x, 444.2, 444.8 were used for diagnosis of PAD [8,10,20,21]. To increase the reliability of the diagnosis, only those with PAD diagnosis for at least three times in the ambulatory claim database or at least once in the inpatient claim database were considered as having PAD.

The SA patients and control subjects were followed from the index dates to either the first diagnosis of PAD, end of the study period, or termination of the record because of death or withdrawal from the insurance program, whichever came first.

### 2.4. Criteria and Definitions of Variables

Comorbidities were identified by the presence of any corresponding diagnostic codes before the index date in the claim databases and they were confirmed by the presence of the codes in at least three ambulatory claims or any inpatient claim. The Charlson Comorbidity Index (CCI) score was calculated based on the comorbidities [8,22].

The CHADS_2_ score was calculated for each subject based on a point system in which one point was assigned for congestive heart failure, hypertension, age ≥75 years, and diabetes mellitus, and two points were assigned for a history of stroke or transient ischemic attack [10]. Modified from the original CHA_2_DS_2_-VASc score, the CHA_2_DS_2_ASc score was calculated for each subject based on a point system in which one point was assigned for congestive heart failure, hypertension, age of 65–74 years, diabetes mellitus, and female sex, and two points were assigned for age ≥75 years and a history of stroke or transient ischemic attack.

### 2.5. Statistical Analysis

For comparing the demographic data, comorbidities, and CCI score between the SA patients and the control subjects, the Pearson’s χ^2^ tests and Student’s *t*-tests were used for categorical variables and continuous variables, respectively. Cumulative incidence of PAD was calculated and compared with the Kaplan–Meier method and log-rank test. To further assess the effect of SA, multivariable Cox proportional hazards regression analyses were performed with the adjustment of sex, age, residency, income level, and comorbidities. In addition to the maximal models, reduced multivariable models were developed with the backward variable selection method, keeping only variables with *p* value less than 0.01, from the maximal model. Hazard ratios (HRs) are presented with 95% confidence intervals (95% CIs). Stratified analyses were also performed by classifying the subjects with sex and age group. The study arms A and B compared the suspected SA patients vs. the control A cohort and the probable SA patients vs. the control B cohort, respectively.

To account for confounding factors, sex, age, residency, income level, and comorbidities were included in a logistic regression model with SA as the dependent variable in order to determine a propensity score. By selecting four propensity score-matched control subjects for each SA patient, propensity score-matched (PM) cohorts were extracted from the original cohorts. As sensitivity analyses to confirm the findings in study arms A and B, the study arms PM-A and PM-B compared the PM-suspected SA patients vs. the control A-PM cohort and the PM-probable SA patients vs. the control B-PM cohort, respectively.

To investigate the role of various scores (such as CHADS_2_ and CHA_2_DS_2_ASc scores) in predicting the risk of PAD in SA patients, Pearson’s χ^2^ tests and Cox proportional hazards regression analyses were used.

To compare the performances of scoring systems in predicting the subsequent PAD risk in SA patients, time-dependent receiver operating characteristic (ROC) curves were used. Time-dependent areas under the curves (AUCs) were summarized and the integrated AUCs were calculated. Using the method of Uno’s concordance estimation, the performances of the scoring systems were pairwise compared.

Extraction and computation of data, data linkage, processing and sampling, and all statistical analyses were performed with the SAS system (version 9.4 for Windows, SAS Institute Inc., Cary, NC, USA). The statistical significance level was set at a two-sided *p*-value of <0.05, unless specified with the analyses.

## 3. Results

### 3.1. Study Population

By the algorithm, 10,702 patients with suspected SA were identified and then matched to 214,040 age- and sex-matched control subjects (control A) for analyses (study arm A), and 4242 probable SA patients and their corresponding control subjects (control B, *n* = 84,840) were extracted for another set of analyses (study arm B) (Figure 1). The baseline characteristics of the study cohorts are presented in Table 1. The mean (± standard deviation (SD)) age of the study population was 47.6 (± 14.8) years and 47.5 (± 13.2) years and 63% and 78% of the study subjects were male in study arms A and B, respectively. As compared with the control subjects, the SA patients had more comorbidities in terms of heart disease, major neurological disorder, chronic pulmonary disease, connective tissue disease, peptic ulcer disease, liver disease, diabetes mellitus, renal disease, and cancer (Table 1).

### 3.2. Similar PAD Risks in SA Patients and Control Subjects

**Figure 2:**
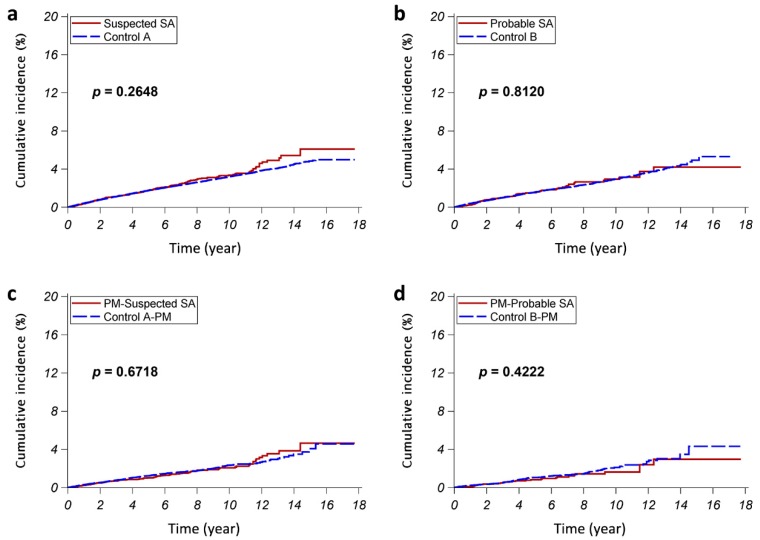
The cumulative incidences of peripheral arterial disease (PAD): The red continuous lines and blue dashed lines show the cumulative incidence of PAD for the sleep apnea (SA) patients and the control subjects, respectively. (**a**) Study arm A (suspected SA vs. control A); (**b**) Study arm B (probable SA vs. control B); (**c**) Study arm PM-A (PM-suspected SA vs. control A-PM); and, (**d**) Study arm PM-B (PM-probable SA vs. control B-PM).

**Figure A1:**
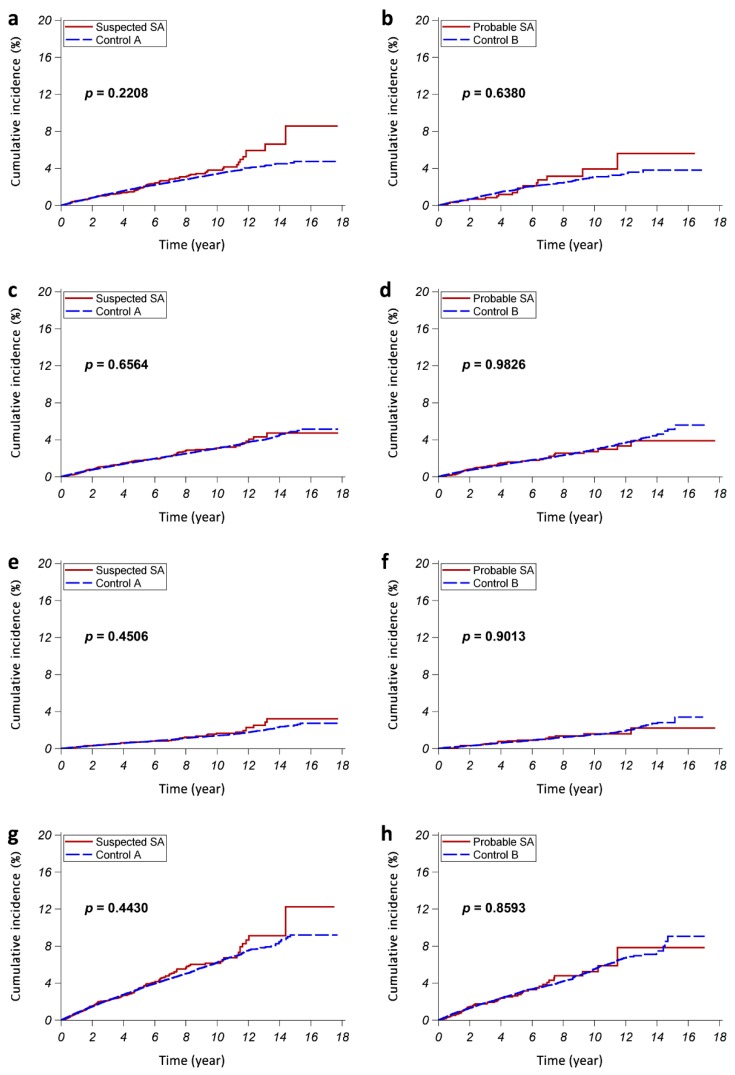
The cumulative incidences of peripheral arterial disease (PAD) in female subjects (**a**,**b**), male subjects (**c**,**d**), subjects ≤50 years of age (**e**,**f**), and subjects >50 years of age (**g**,**h**). The analyses were taken in study arm A (suspected SA vs. control A) (**a**,**c**,**e**,**g**) and study arm B (probable SA vs. control B) (**b**,**d**,**f**,**h**). The red continuous lines and blue dashed lines show the cumulative incidence of PAD for the sleep apnea (SA) patients and the control subjects, respectively.

In both study arms A and B, the SA patients had no statistical difference in the cumulative PAD incidence as compared with the control subjects (both *p >* 0.2) (Figure 2A,B). On stratified analyses, the SA patients had similar cumulative PAD incidence when compared with the corresponding control cohorts in strata of either female subjects, male subjects, subjects with age up to 50 years, or subjects that were older than 50 years (all *p* > 0.2) (Figure A1). On multivariable Cox proportional hazards regression analyses adjusted for sex, age, residency, income, and the presence of various comorbidities, SA was not an independent risk factor for developing PAD (adjusted HR (95% CI): 0.90 (0.79–1.02) in study arm A and 0.81 (0.65–1.03) in study arm B) (Table 2). Stratified analyses showed similar results (Table 2).

To further confirm our findings, we performed sensitivity analyses using propensity score-matched cohorts (Figure 1, Table A1). The cumulative PAD incidence was not statistically different in SA patients as compared with control subjects (both *p >* 0.4) in both study arms PM-A and PM-B (Figure 2C,D). Cox proportional hazards regression analyses revealed that SA was not a risk factor for developing PAD (HR (95%CI): 0.96 (0.78–1.18), *p* = 0.6759 in study arm PM-A; HR (95%CI): 0.86 (0.58–1.25), *p* = 0.6429 in study arm PM-B).

### 3.3. CHADS_2_ and CHA_2_DS_2_ASc Scores Predicts PAD Risks in SA Patients

**Figure 3:**
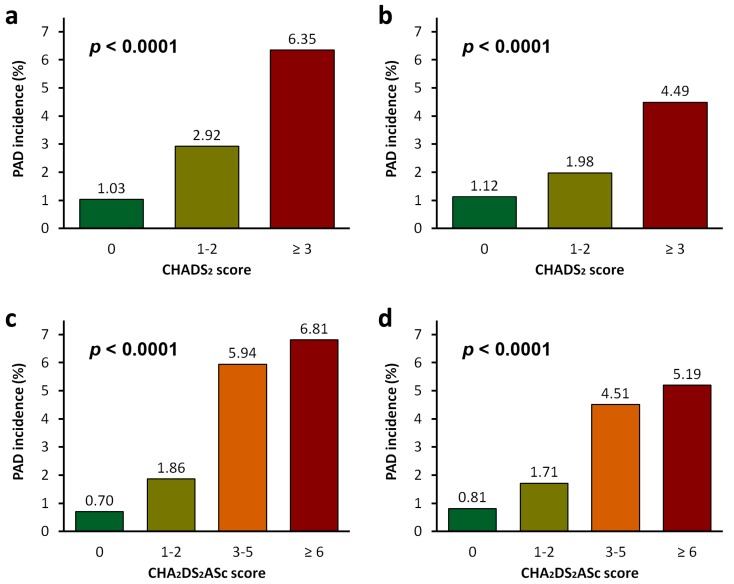
The incidences of peripheral arterial disease (PAD) in (**a**,**c**) suspected sleep apnea (SA) patients and (**b**,**d**) probable SA patients. The CHADS_2_ score was calculated for each subject based on a point system in which one point was assigned for congestive heart failure, hypertension, age ≥75 years, and diabetes mellitus, and two points were assigned for a history of stroke or transient ischemic attack. The CHA_2_DS_2_ASc score was calculated for each subject based on a point system, in which one point was assigned for congestive heart failure, hypertension, age of 65–74 years, diabetes mellitus, and female sex, and two points were assigned for age ≥75 years and a history of stroke or transient ischemic attack.

**Figure 4:**
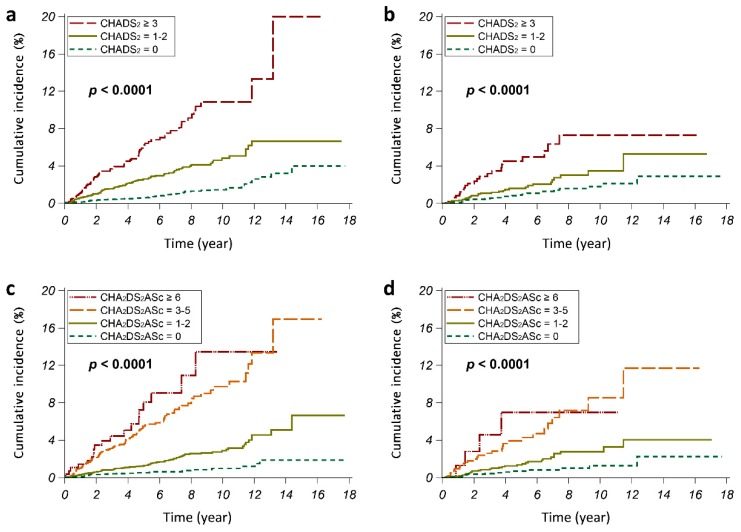
The cumulative incidences of peripheral arterial disease (PAD) in (**a**,**c**) suspected sleep apnea (SA) patients and (**b**,**d**) probable SA patients. The CHADS_2_ score was calculated for each subject based on a point system in which one point was assigned for congestive heart failure, hypertension, age ≥75 years, and diabetes mellitus, and two points were assigned for a history of stroke or transient ischemic attack. The CHA_2_DS_2_ASc score was calculated for each subject based on a point system, in which one point was assigned for congestive heart failure, hypertension, age of 65–74 years, diabetes mellitus, and female sex, and two points were assigned for age ≥75 years and a history of stroke or transient ischemic attack.

CHADS_2_ and CHA_2_DS_2_ASc scores were calculated for each SA patient. The incidence of PAD was significantly higher in SA patients with higher CHADS_2_ and CHA_2_DS_2_ASc scores (all *p* < 0.0001) (Figure 3). In either suspected SA or probable SA patients, those with a CHADS_2_ score of 0 and a CHA_2_DS_2_ASc score of 0 had a PAD incidence of less than 1.2%, whereas those with a CHADS_2_ score of ≥3 and a CHA_2_DS_2_ASc score of ≥6 had PAD incidence more than 4.4%. The cumulative incidence of PAD was also significantly higher in SA patients with higher CHADS_2_ and CHA_2_DS_2_ASc scores (all *p* < 0.0001) (Figure 4). Using Cox regression analyses, we found that SA patients with higher CHADS_2_ and CHA_2_DS_2_ASc scores had higher risk for developing PAD (Table 3). In the suspected SA cohort, patients with CHADS_2_ score of ≥3 had significantly increased risk for developing PAD than those with a CHADS_2_ score of 0 (HR (95% CI): 7.68 (5.47–10.77)); patients with CHA_2_DS_2_ASc score of ≥6 also had significantly increased risk for developing PAD than those with CHA_2_DS_2_ASc score of 0 (HR (95% CI): 13.76 (7.60–24.92)). Analyses of the probable SA cohort showed consistent results.

### 3.4. New Scoring System Predicting PAD Risks in SA Patients

**Figure 5:**
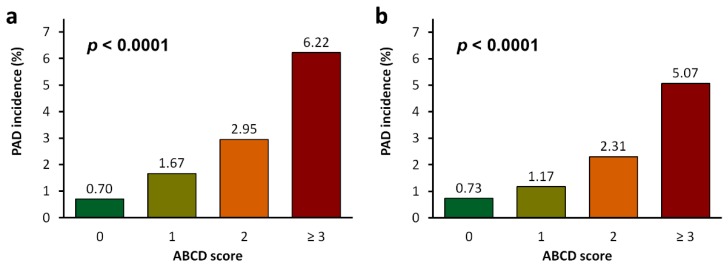
The incidences of peripheral arterial disease (PAD) in (**a**) suspected sleep apnea (SA) patients and (**b**) probable SA patients. The ABCD (Age, Blood pressure, Cerebral vascular disease, Diabetes mellitus) score was calculated for each subject based on a point system in which one point was assigned for age >50 years, high blood pressure (hypertension), cerebral vascular disease, and diabetes mellitus, and an extra point was given for age >65 years.

In addition to the CHADS_2_ and CHA_2_DS_2_ASc scores, we tried to pursue a more accurate model by using multivariable Cox regression models (Table A2). The reduced multivariable model identified age, hypertension, cerebral vascular disease, and diabetes as the most significant and independent risk factors for incident PAD in suspected SA patients. We therefore proposed the “ABCD score”, which assigned a point for **A**ge > 50 years and an extra point for age > 65 years, a point for high **B**lood pressure (hypertension), a point for **C**erebral vascular disease, and a point for **D**iabetes mellitus. As shown in Figure 5, the incidence of PAD was significantly higher in SA patients with a higher ABCD score (*p* < 0.0001 in both patients with suspected SA and those with probable SA). The SA patients with an ABCD score of ≥3 had a significantly increased risk for developing PAD than those with an ABCD score of 0 (HR (95% CI): 3.93 (2.83–5.47) in the suspected SA cohort and 8.57 (4.52–16.24) in the probable SA cohort) (Table 4).

**Figure A2:**
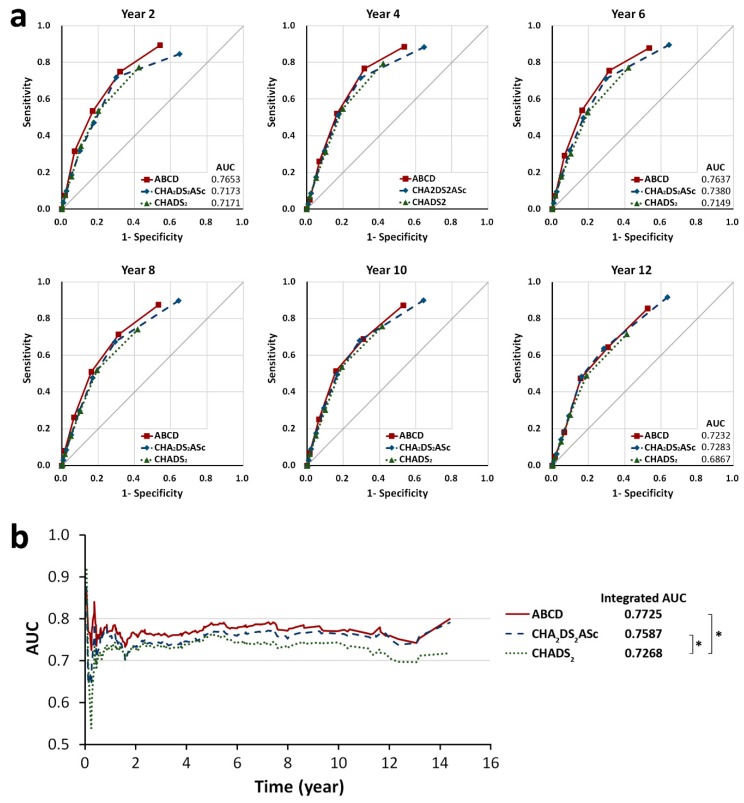
(**a**) The time-dependent receiver operating characteristic (ROC) curves used to illustrate the performances of scoring systems, including CHADS_2_, CHA_2_DS_2_ASc, and ABCD scores, in predicting subsequent peripheral arterial disease (PAD) in suspected sleep apnea patients. (**b**) Time-dependent area under the curves (AUCs) and the integrated AUCs. * *p* < 0.05, pairwise comparison using the method of Uno’s concordance estimation.

**Figure A3:**
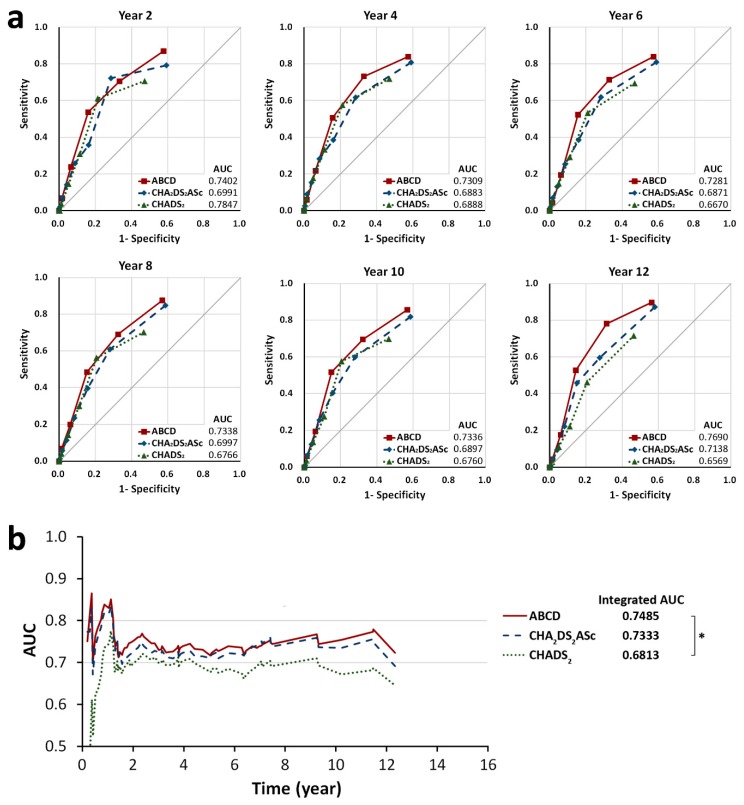
(**a**) The time-dependent receiver operating characteristic (ROC) curves used to illustrate the performances of scoring systems, including CHADS_2_, CHA_2_DS_2_ASc, and ABCD scores, in predicting subsequent peripheral arterial disease (PAD) in probable sleep apnea patients. (**b**) Time-dependent area under the curves (AUCs) and the integrated AUCs. * *p* < 0.05, pairwise comparison using the method of Uno’s concordance estimation.

To determine whether the ABCD score had better performance than CHADS_2_ and CHA_2_DS_2_ASc scores in predicting incident PAD in SA patients, time-dependent ROC curves of these scoring systems were compared (Figure A2). In the cohort of suspected SA patients, the integrated time-dependent AUC of ABCD (0.7725) was similar to that of CHA_2_DS_2_ASc score (0.7587), but was significantly higher than that of CHADS_2_ score (0.7268) (Figure A2b). The analyses using the cohort of probable SA patients showed consistent results (Figure A3).

## 4. Discussion

This large population-based cohort study revealed that patients with SA had a similar incidence of PAD as subjects without SA. In addition to the analyses using SA patients identified with merely the diagnostic codes (suspected SA group), we also performed analyses using patients having SA diagnosis after PSG (probable SA group) and found similar results. The analyses of the propensity score-matched cohorts also showed similar results. Furthermore, we identified that both CHADS_2_ and CHA_2_DS_2_ASc scores, as well as a newly-proposed ABCD score, were good predictors for incident PAD in SA patients.

A few observational and epidemiological studies have reported the association between SA and PAD. A research group in Bonn, Germany has found a high prevalence rate of SA (78%) in 91 cases of confirmed lower extremity artery disease [23]. They also found a similar prevalence rate of SA (81.4%) in a cohort of 59 patients who already had PAD undergoing percutaneous revascularization [24]. On the other hand, the prevalence of PAD was as high as 88% in the suspected OSA group and it was even higher (98%) among the confirmed OSA cases [7]. In another large-scale observational cohort study with 5365 participants, subsequent PAD incidence was significantly higher in patients with self-reported SA (adjusted hazard ratio (95% CI): 1.93 (1.05–3.53)) [25].

Some pathophysiological mechanisms may link sleep disturbance and atherosclerosis, including insulin resistance, endothelial dysfunction, hypertension, and inflammation [25]. OSA patients may present frequent arousals and sleep deprivation, negative intrathoracic pressure, hypercapnia, and repeated deoxygenation and re-oxygenation. These may cause sympathetic activation, metabolic dysregulation, left atrial enlargement, systemic inflammation, hypercoagulability, and endothelial dysfunction, resulting in the future development of cardiovascular and cerebrovascular diseases, such as hypertension, coronary artery disease, heart failure, atrial fibrillation, and stroke [26].

However, SA shared much comorbidity with PAD, which may confound the statistical analyses. In a previous case-control study using NHIRD, Chen et al. reported that PAD was significantly associated with SA (odds ratio (95% CI): 1.6 (1.25–2.04), *p* < 0.001) in the univariate logistic regression analysis [8]. However, the association was attenuated and it became insignificant after adjusting for multiple comorbidities, including hypertension, diabetes, coronary artery disease or myocardial infarction, chronic kidney disease, hyperlipidemia, hyperuricemia, and obesity (adjusted odds ratio (95% CI): 1.26 (0.98–1.62), *p* = 0.075). In contrast to their case-control study design with logistic regression analyses, our study used cohort study design with Cox regression analyses, which took the timeframe between SA and incident PAD into consideration. Besides, each case was matched to three controls with sex and 10-year age interval, and index year in their study, whereas each case was matched to 20 controls with sex and age (precisely the year) in our study. We believe that our study design has better opportunity to evaluate the association between SA and PAD more precisely. In contrast to their study results, we found that SA patients did not have significantly increased risk of subsequent PAD.

CHADS_2_ and CHA_2_DS_2_-VASc scores were originally developed to predict the risk of stroke in patients with non-valvular atrial fibrillation, and to guide the treatment strategy to prevent stroke. Recent studies had extended their utility to predict peripheral vascular events, even in patients without atrial fibrillation. Hsu et al. reported that CHADS_2_ score has good correlation with ankle-brachial index (ABI) <0.9 and it has good performance in predicting the risk of new onset PAD in patients without atrial fibrillation [10,11]. They also found that a modified CHA_2_DS_2_-VASc score, excluding peripheral arterial occlusive disease from the original vascular disease item, was significantly associated with ABI <0.9 [27]. In line with the studies by Hsu et al., we found that the risk of PAD was significantly increased with increasing CHADS_2_ and CHA_2_DS_2_ASc scores in SA patients. Calculating these scores may help in stratifying the risk of PAD in SA patients. In this study, we further developed a simpler scoring system, the “ABCD score”, which showed significantly better performance in predicting the PAD risk in SA patients than did the CHADS_2_ score. Due to its simplicity, the ABCD score may be widely adopted in clinical settings.

Our study has some limitations. Firstly, a commonplace limitation of using a claim-based registry database is the inaccuracy that is brought about by identifying patients based only on diagnostic codes, instead of standardized criteria that are used in clinical trials. Although the claims of disease-specific treatment might be used to confirm the diagnosis in studies using claim databases, the main SA-specific treatment, continuous positive airway pressure, was not covered by NHI. Therefore, in addition to the “suspected SA” cohort, which enrolled SA patients based on diagnostic codes, we attempted to improve the accuracy by further extracting the “probable SA” cohort, which only included those having SA diagnosis after PSG. The analyses using both cohorts were consistent. Although we could not obtain the ABI of each patient from our database, we tried to increase the accuracy of PAD diagnosis by ensuring the presence of the diagnostic codes in at least one inpatient claim or three ambulatory claims. Secondly, there might be some patients with undiagnosed SA in the control group, which might lead to underestimating the effect of SA on PAD incidence. However, this would be unlikely to change the final conclusion of this study that SA was not an independent risk factor of PAD, because of the insignificantly slightly decreased the risk of PAD in SA patients shown in the current study (hazard ratios of ≤0.9). Nevertheless, this posits the cautious interpretation of our results. Thirdly, obstructive SA and central SA (CSA) were undistinguishable based on the diagnostic codes in our database, hence it is difficult to evaluate whether OSA and CSA affect the risk of PAD in the same way. However, because over 90% of patients of SA are OSA, it is OSA that might substantially contribute to the result [1]. Fourthly, we could not obtain information regarding the severity of SA and PAD in the database, so we could not further investigate whether SA severity influenced future PAD risks. Fifthly, some well-known risk factors of PAD, including obesity, cigarette smoking, and blood pressure level, as well as the SA-specific treatment, especially continuous positive airway pressure, could not be identified from the database. We therefore used CCI to surrogate these confounding factors. Nevertheless, bias might still exist while using this method and caution should be taken when interpreting our results.

## 5. Conclusions

In conclusion, the present large nationwide population-based cohort study determined that SA was not an independent risk factor for PAD. CHADS_2_ and CHA_2_DS_2_ASc scores, as well as the newly proposed “ABCD score” might be useful in predicting the future risk of PAD in SA patients. Further large-scale, prospective, and longitudinal studies are needed to confirm our findings and to elucidate any causative association between SA and PAD.

## Figures and Tables

**Table 1:** Baseline characteristics of the study population.

	Study Arm A			Study Arm B		
	Suspected SA	Control A	*p*-Value	Probable SA	Control B	*p*-Value
*n*	10,702	214,040		4242	84,840	
Sex, *n* (%)						
Female	3967 (37%)	79,340 (37%)		932 (22%)	18,640 (22%)	
Male	6735 (63%)	134,700 (63%)		3310 (78%)	66,200 (78%)	
Age (year), mean ± SD	47.6 ± 14.8	47.6 ± 14.8	>0.99	47.5 ± 13.2	47.5 ± 13.2	>0.99
Age (year), *n* (%)			>0.99			>0.99
≤40	3668 (34%)	73,360 (34%)		1348 (32%)	26,960 (32%)	
40 < age ≤ 50	2669 (25%)	53,380 (25%)		1190 (28%)	23,800 (28%)	
>50	4365 (41%)	87,300 (41%)		1704 (40%)	34,080 (40%)	
Residency			<0.0001			<0.0001
Northern Taiwan	4912 (46%)	106,227 (50%)		1825 (43%)	41,908 (49%)	
Other areas	5790 (54%)	107,813 (50%)		2417 (57%)	42,932 (51%)	
Monthly income (NT$), *n* (%)			<0.0001			<0.0001
≤24,000	6140 (57%)	135,967 (64%)		2065 (49%)	50,746 (60%)	
>24,000	4562 (43%)	78,073 (36%)		2177 (51%)	34,094 (40%)	
CCI score, mean ± SD	1.5 ± 1.9	0.9 ± 1.6	<0.0001	1.5 ± 1.8	0.9 ± 1.5	<0.0001
CCI score, *n* (%)			<0.0001			<0.0001
= 0	4203 (39%)	124,704 (58%)		1581 (37%)	49,278 (58%)	
= 1	2723 (25%)	43,992 (21%)		1121 (26%)	17,941 (21%)	
≥2	3776 (35%)	45,344 (21%)		1540 (36%)	17,621 (21%)	
Underlying diseases, *n* (%)						
Heart disease	519 (5%)	5293 (2%)	<0.0001	211 (5%)	1819 (2%)	<0.0001
Myocardial infarction	138 (1%)	1552 (1%)	<0.0001	63 (1%)	613 (1%)	<0.0001
Congestive heart failure	416 (4%)	4206 (2%)	<0.0001	165 (4%)	1371 (2%)	<0.0001
Peripheral vascular disease	57 (1%)	668 (0%)	<0.0001	21 (0%)	279 (0%)	0.0682
Major neurological disorder	1148 (11%)	13,217 (6%)	<0.0001	496 (12%)	4661 (5%)	<0.0001
Cerebral vascular disease	1087 (10%)	12,379 (6%)	<0.0001	474 (11%)	4404 (5%)	<0.0001
Dementia	118 (1%)	1570 (1%)	<0.0001	46 (1%)	423 (0%)	<0.0001
Hemiplegia	89 (1%)	1590 (1%)	0.2980	35 (1%)	619 (1%)	0.4772
Chronic pulmonary disease	2956 (28%)	33,877 (16%)	<0.0001	1237 (29%)	12,803 (15%)	<0.0001
Connective tissue disease	233 (2%)	2656 (1%)	<0.0001	82 (2%)	907 (1%)	<0.0001
Peptic ulcer disease	3387 (32%)	41,937 (20%)	<0.0001	1354 (32%)	16,613 (20%)	<0.0001
Liver disease	2302 (22%)	26,786 (13%)	<0.0001	1003 (24%)	11,462 (14%)	<0.0001
Diabetes mellitus	1387 (13%)	20,632 (10%)	<0.0001	584 (14%)	8118 (10%)	<0.0001
Renal disease	451 (4%)	5501 (3%)	<0.0001	192 (5%)	2079 (2%)	<0.0001
Cancer	578 (5%)	7524 (4%)	<0.0001	191 (5%)	2828 (3%)	<0.0001

Abbreviation: SA = sleep apnea; CCI = Charlson Comorbidity Index; SD = standard deviation.

**Table 2:** Multivariable Cox regression analyses assessing the effect of sleep apnea (SA) on the risk of developing peripheral arterial disease (PAD).

	Study arm A Suspected SA		Study arm B Probable SA	
	HR (95%CI)	*p*-Value	HR (95%CI)	*p*-Value
All patients	0.1093	0.81 (0.65–1.03)	0.90 (0.79–1.02)	0.0838
Stratified analyses				
Female	0.96 (0.78–1.18)	0.6916	0.90 (0.56–1.45)	0.6602
Male	0.85 (0.72–1.01)	0.0695	0.79 (0.61–1.04)	0.0880
Age ≤ 50	0.84 (0.65–1.08)	0.1804	0.81 (0.54–1.23)	0.3246
Age > 50	0.89 (0.77–1.04)	0.1598	0.81 (0.61–1.07)	0.1337

Multivariable Cox regression analyses were adjusted for sex, age, residency, income level, and comorbidities, except for the variables used for stratification. Adjusted hazard ratios (HRs) with 95% confidence intervals (CIs) of sleep apnea (SA) are presented.

**Table 3:** Risks of developing peripheral arterial disease (PAD) in sleep apnea (SA) patients with different risk scores.

Score	Suspected SA		Probable SA	
	HR (95%CI)	*p*-Value	HR (95%CI)	*p*-Value
CHADS_2_				
0	1.00		1.00	
1–2	3.18 (2.32–4.36)	<0.0001	1.90 (1.12–3.23)	0.0179
≥ 3	7.68 (5.47–10.77)	<0.0001	4.76 (2.68–8.45)	<0.0001
CHA_2_DS_2_ASc				
0	1.00		1.00	
1-2	2.83 (1.83–4.37)	<0.0001	2.27 (1.21–4.28)	0.0108
3-5	9.95 (6.45–15.35)	<0.0001	6.56 (3.45–12.48)	<0.0001
≥6	13.76 (7.60–24.92)	<0.0001	9.37 (3.07–28.57)	<0.0001

The CHADS_2_ score was calculated for each subject based on a point system in which one point was assigned for congestive heart failure, hypertension, age ≥75 years, and diabetes mellitus, and two points were assigned for a history of stroke or transient ischemic attack. The CHA_2_DS_2_ASc score was calculated for each subject based on a point system, in which one point was assigned for congestive heart failure, hypertension, age of 65–74 years, diabetes mellitus, and female sex, and two points were assigned for age ≥75 years and a history of stroke or transient ischemic attack. Abbreviation: SA = sleep apnea; HR = hazard ratio; CI = confidence interval.

**Table 4:** Risks of developing peripheral arterial disease (PAD) in sleep apnea (SA) patients with different ABCD scores.

Score	Suspected SA		Probable SA	
	HR (95%CI)	*p*-Value	HR (95%CI)	*p*-Value
ABCD				
0	1.00		1.00	
1	1.11 (0.76–1.61)	0.6027	1.69 (0.77–3.69)	0.1927
2	2.07 (1.46–2.94)	<0.0001	3.46 (1.68–7.13)	0.0008
≥3	3.93 (2.83–5.47)	<0.0001	8.57 (4.52–16.24)	<0.0001

The ABCD score was calculated for each subject based on a point system in which one point was assigned for age >50 years, high blood pressure (hypertension), cerebral vascular disease, and diabetes mellitus, and an extra point was given for age >65 years. Abbreviation: SA = sleep apnea; HR = hazard ratio; CI = confidence interval.

## Appendix A

**Table A1:** Baseline characteristics of the propensity score-matched (PM) cohorts.

	Study Arm PM-A			Study Arm PM-B		
	PM-Suspected SA	Control A-PM	*p*-Value	PM-Probable SA	Control B-PM	*p*-Value
*n*	7985	31,940		3164	12,656	
Sex, *n* (%)						
Female	2837 (36%)	11,348 (36%)		637 (20%)	2548 (20%)	
Male	5148 (64%)	20,592 (64%)		2527 (80%)	10,108 (80%)	
Age (year), mean ± SD	44.5 ± 13.3	44.5 ± 13.3	>0.99	44.7 ± 11.9	44.7 ± 11.8	>0.99
Age (year), *n* (%)			>0.99			>0.99
≤40	3258 (41%)	13,013 (41%)		1203 (38%)	4804 (38%)	
40 < age ≤ 50	2143 (27%)	8588 (27%)		958 (30%)	3842 (30%)	
>50	2584 (32%)	10,339 (32%)		1003 (32%)	4010 (32%)	
Residency			0.5303			0.585
Northern Taiwan	3647 (46%)	14,713 (46%)		1345 (43%)	5448 (43%)	
Other areas	4338 (54%)	17,227 (54%)		1819 (57%)	7208 (57%)	
Monthly income (NT$), *n* (%)			0.9639			0.9427
≤24,000	4342 (54%)	17,359 (54%)		1427 (45%)	5699 (45%)	
>24,000	3643 (46%)	14,581 (46%)		1737 (55%)	6957 (55%)	
CCI score, mean ± SD	0.7 ± 0.9	0.7 ± 0.9	>0.99	0.7 ± 0.9	0.7 ± 0.9	>0.99
CCI score, *n* (%)			>0.99			>0.99
= 0	4199 (53%)	16,796 (53%)		1580 (50%)	6320 (50%)	
= 1	2499 (31%)	9996 (31%)		1037 (33%)	4148 (33%)	
≥ 2	1287 (16%)	5148 (16%)		547 (17%)	2188 (17%)	
Underlying diseases, *n* (%)						
Heart disease	12 (0%)	48 (0%)	>0.99	7 (0%)	28 (0%)	>0.99
Myocardial infarction	3 (0%)	24 (0%)	0.2481	1 (0%)	17 (0%)	0.1253
Congestive heart failure	9 (0%)	24 (0%)	0.2961	6 (0%)	11 (0%)	0.1147
Peripheral vascular disease	0 (%)	0 (%)	0.2961	0 (%)	0 (%)	0.1147
Major neurological disorder	190 (2%)	760 (2%)	>0.99	76 (2%)	304 (2%)	>0.99
Cerebral vascular disease	190 (2%)	757 (2%)	0.9607	76 (2%)	304 (2%)	>0.99
Dementia	0 (0%)	3 (0%)	0.3865	0 (%)	0 (%)	>0.99
Hemiplegia	1 (0%)	4 (0%)	>0.99	1 (0%)	4 (0%)	>0.99
Chronic pulmonary disease	1519 (19%)	6076 (19%)	>0.99	657 (21%)	2628 (21%)	>0.99
Connective tissue disease	12 (0%)	48 (0%)	>0.99	3 (0%)	12 (0%)	>0.99
Peptic ulcer disease	1903 (24%)	7612 (24%)	>0.99	770 (24%)	3080 (24%)	>0.99
Liver disease	1189 (15%)	4756 (15%)	>0.99	530 (17%)	2120 (17%)	>0.99
Diabetes mellitus	345 (4%)	1380 (4%)	>0.99	151 (5%)	604 (5%)	>0.99
Renal disease	23 (0%)	92 (0%)	>0.99	7 (0%)	28 (0%)	>0.99
Cancer	72 (1%)	286 (1%)	0.9577	26 (1%)	104 (1%)	>0.99

Abbreviation: SA = sleep apnea; CCI = Charlson Comorbidity Index; SD = standard deviation.

**Table A2:** Multivariable Cox regression analyses investigating the risk factors of incident peripheral arterial disease (PAD).

	Suspected SA				Probable SA				Proposed ABCD Score
	Maximal Model		Reduced Model ^†^		Maximal Model		Reduced Model ^†^		
	HR (95%CI)	*p*-Value	HR (95%CI)	*p*-Value	HR (95%CI)	*p*-Value	HR (95%CI)	*p*-Value	
Male (vs. female)	1.00 (0.77–1.30)	0.9909			1.07 (0.62–1.86)	0.7985			
Age (year) (vs. ≤ 50):									
50 < age ≤ 65	2.07 (1.45–2.96)	<0.0001	2.25 (1.59–3.20)	<0.0001	2.09 (1.16–3.77)	0.0142	2.60 (1.52–4.48)	0.0005	**1**
>65	3.13 (2.07–4.74)	<0.0001	3.95 (2.70–5.79)	<0.0001	4.08 (1.93–8.62)	0.0002	7.17 (4.07–12.63)	<0.0001	**2**
Residency in Northern Taiwan (vs. other areas)	0.80 (0.62–1.04)	0.0950			0.80 (0.51–1.26)	0.3356			
Monthly income (NT$) > 24,000 (≤24,000)	0.74 (0.53–1.02)	0.0660			0.83 (0.49–1.40)	0.4757			
Underlying diseases (with vs. without):									
Hypertension	1.62 (1.18–2.22)	0.0029	1.70 (1.24–2.32)	0.0009	1.18 (0.68–2.02)	0.5584			**1**
Myocardial infarction	1.28 (0.62–2.63)	0.5027			1.10 (0.26–4.63)	0.8991			
Congestive heart failure	0.84 (0.52–1.36)	0.4794			1.10 (0.48–2.53)	0.8252			
Cerebral vascular disease	1.52 (1.10–2.11)	0.0123	1.61 (1.17–2.21)	0.0031	1.44 (0.79–2.65)	0.2372			**1**
Dementia	1.20 (0.55–2.62)	0.6415			2.27 (0.77–6.71)	0.1382			
Hemiplegia	0.87 (0.35–2.17)	0.7655			1.56 (0.45–5.44)	0.4849			
Chronic pulmonary disease	1.39 (1.05–1.84)	0.0214			1.04 (0.63–1.72)	0.8743			
Connective tissue disease	1.42 (0.72–2.78)	0.3110			1.04 (0.25–4.29)	0.9612			
Peptic ulcer disease	1.24 (0.94–1.63)	0.1358			1.22 (0.75–1.99)	0.4271			
Liver disease	0.89 (0.65–1.21)	0.4495			0.86 (0.49–1.48)	0.5811			
Diabetes mellitus	1.74 (1.28–2.36)	0.0003	1.74 (1.29–2.34)	0.0003	1.69 (0.98–2.94)	0.0608			**1**
Renal disease	1.35 (0.88–2.08)	0.1742			1.36 (0.63-2.93)	0.4376			
Cancer	1.00 (0.77–1.30)	0.9909			‡	0.9766			

HR = hazard ratio; CI = confidence interval; † Reduced multivariable models was developed with backward variable selection method, keeping only variables with *p* value less than 0.01, from the maximal model. ‡ Due to relatively small sample size, the hazard ratio could not be estimated. This variable was therefore not included in the models.
